# Geometric data of commercially available tricuspid valve annuloplasty devices

**DOI:** 10.1016/j.dib.2024.110051

**Published:** 2024-01-09

**Authors:** Collin E. Haese, Mrudang Mathur, Marcin Malinowski, Tomasz A. Timek, Manuel K. Rausch

**Affiliations:** a Walker Department of Mechanical Engineering, The University of Texas at Austin, 204 E Dean Keeton St, Austin, 78712, TX, USA; b Department of Cardiac Surgery, Medical University of Silesia in Katowice, 15 Poniatowskiego, 40-055 Katowice, Poland; c Division of Cardiothoracic Surgery, Spectrum Health, 221 Michigan St NE, Suite 300, Grand Rapids, 49503, MI, USA; d Department of Aerospace Engineering and Engineering Mechanics, The University of Texas at Austin, 2617 Wichita St North Office Building A, Austin, 78712, TX, USA; e Department of Biomedical Engineering, The University of Texas at Austin, 107W Dean Keeton St, Austin, 78712, TX, USA; f Oden Institute for Computational Engineering and Sciences, The University of Texas at Austin, 201 E 24th St, Austin, 78712, TX, USA

**Keywords:** Repair, Three-dimensional scan, Micro-computed tomography, Downsizing, Annulus

## Abstract

Tricuspid valve annuloplasty is the gold standard surgical treatment for functional tricuspid valve regurgitation. During this procedure, ring-like devices are implanted to reshape the diseased tricuspid valve annulus and to restore function. For the procedure, surgeons can choose from multiple available device options varying in shape and size. In this article, we provide the three-dimensional (3D) scanned geometry (*.stl) and reduced midline (*.vtk) of five different annuloplasty devices of all commercially available sizes. Three-dimensional images were captured using a 3D scanner. After extracting the surface geometry from these images, the images were converted to 3D point clouds and skeletonized to generate a 3D midline of each device. In total, we provide 30 data sets comprising the Edwards Classic, Edwards MC3, Edwards Physio, Medtronic TriAd, and Medtronic Contour 3D of sizes 26–36. This dataset can be used in computational models of tricuspid valve annuloplasty repair to inform accurate repair geometry and boundary conditions. Additionally, others can use these data to compare and inspire new device shapes and sizes.

Specifications table

**Table:** 

Subject	Biomedical Engineering
Specific subject area	Biomedical Devices, Biomedical Engineering, Mechanical Engineering, and Computational Mechanics
Data format	Raw, Analyzed
Type of data	Geometry File, Reduced Geometry File
Data collection	Three-dimensional scanning was used to characterize all available sizes of three Edwards Lifesciences (Irvine, CA) and two Medtronic (Minneapolis, MN) tricuspid valve annuloplasty devices.
Data source location	Institution: The University of Texas at Austin City/State: Austin, Texas Country: United States of America
Data accessibility	All data are available with the article. Repository name: Texas Data Repository Data identification number: 3D Scans [1]: https://doi.org/10.18738/T8/EFU2MA Midline Geometries [2]: https://doi.org/10.18738/T8/7SOWNX Direct URL to data: https://dataverse.tdl.org/dataverse/tricuspidannuloplastydevices Instructions for accessing these data: All files are available for download through the open access Soft Tissue Biomechanics Laboratory Dataverse hosted on the Texas Data Repository.
Related research article	Mathur, M., Malinowski, M., Timek, T. A. & Rausch, M. K. Tricuspid Annuloplasty Rings: A Quantitative Comparison of Size, Nonplanar Shape, and Stiffness. The Annals of Thoracic Surgery 110, 1605–1614 (2020). URL: https://linkinghub.elsevier.com/retrieve/pii/S0003497520304409. [3]

## 1 Value of the Data

• The geometric data of these tricuspid annuloplasty devices will allow surgeons to objectively compare these devices and thus help make the best selection for tricuspid valve repair.
• These data may further help scientists who build computational models of the tricuspid valve and tricuspid valve repair procedures [4], [5], [6], [7], [8], [9], [10], [11], [12]. For example, predictive models of tricuspid valve annuloplasty procedures may use our data to inform their device geometries.
• Finally, engineers may use these data to inspire new device designs. That is, they may use the existing geometries (and morphological metrics) as a starting point for their design efforts.

## 2 Background

The tricuspid valve ensures unidirectional blood flow through the right atrium and ventricle. As a result of primary diseases, often on the left side of the heart, the valve can become ineffective and leak, i.e., regurgitate [13,14]. Annuloplasty repair is one of the most common surgical procedures to treat functional tricuspid regurgitation [15,16], and several clinical studies have shown improved patient outcomes with treatment via annuloplasty devices [17], [18], [19], [20]. We direct the reader to these studies for a thorough clinical discussion of tricuspid valve repair. Annuloplasty devices’ fundamental function is to eliminate leakage by restoring healthy tricuspid valve shape and coaptation. Currently, surgeons can select from many device shapes and sizes. A table providing a quantitative comparison between all available annuloplasty device shapes and sizes considered in this dataset is provided in the companion article “Tricuspid Annuloplasty Rings: A Quantitative Comparison of Size, Nonplanar Shape, and Stiffness” by Mathur and colleagues [3]. We also direct the reader to a recent study in which we investigated the impact of device shape and size on tricuspid valve mechanics as one example of how our data may be useful [4].

## 3 Data Description

The tricuspid annuloplasty device dataset is split into two primary sections: device 3D scans [1] and device midlines [2]. Each section contains six sizes of all five types of annuloplasty devices, namely 30 device 3D scans and 30 device midlines. The 3D scan files are “*.stl” files, while the device midlines are ParaView Visualization Toolkit “*.vtk” legacy files. Files are named as “Device_Type_Device_Size.*” throughout.

The midline data is structured in the *.vtk file as follows: the three-dimensional coordinates of the midline points are listed under the heading “POINTS number_of_points float” with three columns representing the x, y, and z coordinates, and the number of rows matches the number of points. The scalar coloring data is listed under the heading “SCALARS name_of_scalar_data float” as a single column of scalar values with the number of rows again matching the number of midline points. We include Height, 2DCurvature, and 3DCurvature as scalar data to choose from. Lastly, a “README.pdf” file is included in the dataset which describes the data and provides helpful suggestions for how to view the midline geometries in ParaView.

## 4 Experimental Design, Materials and Methods

**Fig. 1:**
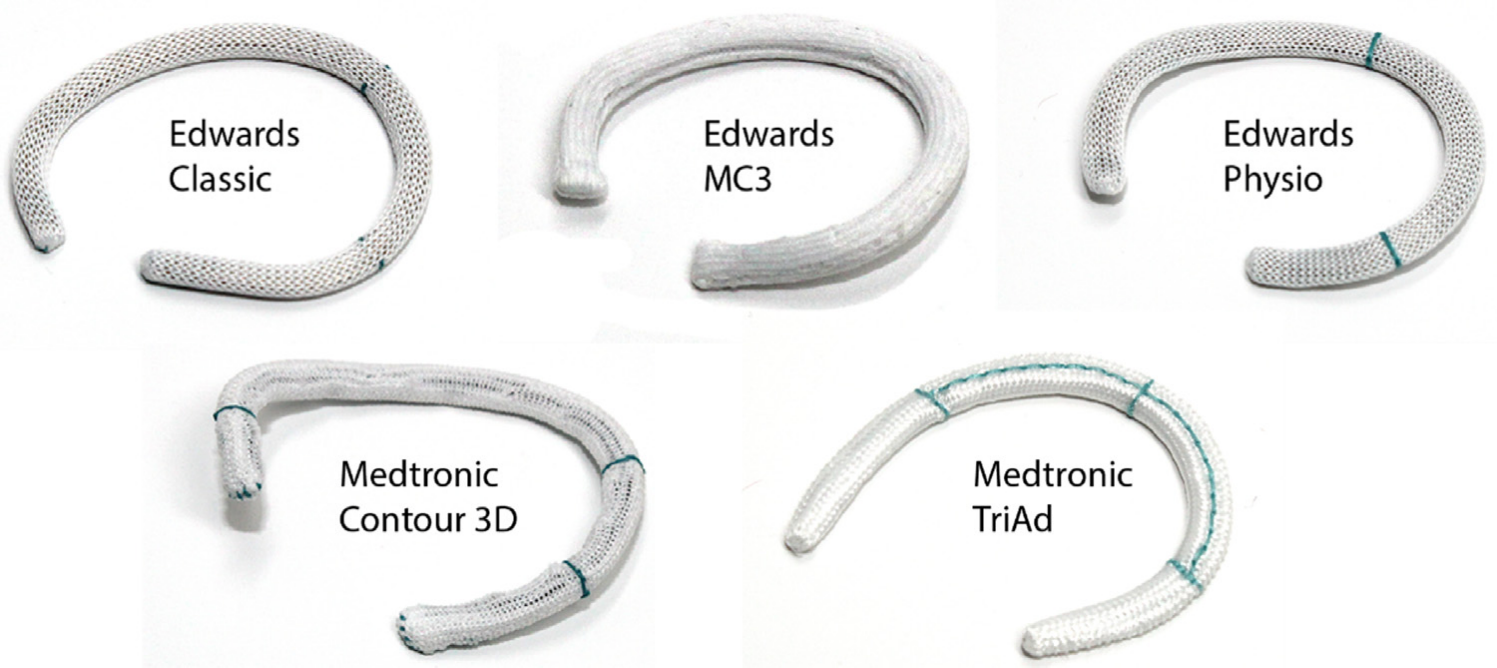
The five tricuspid annuloplasty devices considered in this dataset. Namely, the Carpentier-Edwards Classic device model 4500, the Edwards MC3 Tricuspid device model 4900, the Carpentier-Edwards Physio Tricuspid device model 6200, the Medtronic Contour 3D device model 690R, and the Medtronic TriAd Adams Band model 900 SFC.

**Fig. 2:**
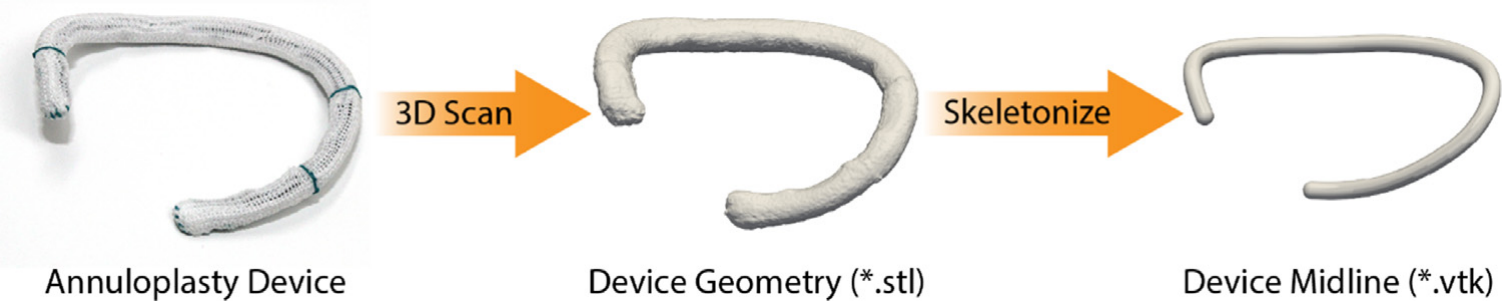
Annuloplasty device characterization pipeline of the dataset, where 3D scans of the devices are reduced to a midline profile.

We captured five device types of six sizes each. The five device types are shown in Fig. 1. Three devices were from Edwards Lifesciences (Irvine, CA): the Carpentier-Edwards Classic device model 4500 (Classic), the Carpentier-Edwards Physio Tricuspid device model 6200 (Physio), and the Edwards MC3 Tricuspid device model 4900 (MC3). Two devices were from Medtronic (Minneapolis, MN): the Medtronic TriAd Adams Band model 900 SFC (TriAd) and the Medtronic Contour 3D device model 690R (Contour). All devices were available in sizes 26, 28, 30, 32, 34, or 36. We scanned each device using a NextEngine Ultra HD 3D Scanner (Santa Monica, CA) and saved their surface representations in the respective “*.stl” files. We then reduced these surface representations to 3D point clouds for the morphometric characterization of each device. Subsequently, we used a custom skeletonization algorithm in MATLAB (2019a, Mathworks, Natick, MA) to create a three-dimensional midline of the annuloplasty device from its 3D point cloud. The midline represents the centroidal axis of the annuloplasty device. A visual summary of the device characterization pipeline is presented in Fig. 2.

Additionally, three morphological metrics are provided as embedded data in the ParaView midline files: 2D curvature, 3D curvature, and height. A standard formula based on the first and second derivatives of the best-fit spline with respect to the arc-length parameter were used to compute the 2D and 3D curvature [21], [22], [23]. Two-dimensional curvature is defined within the least-squares plane of the device. Height was computed as the orthogonal distance between each midline point and the least-squares plane of the midline geometry. Additional geometric metrics, such as diameter, perimeter, and area, can be computed using the device's midline data. For more details on how each metric was computed, please see our prior publications [3,[24], [25], [26]].

## Limitations

The data presented herein represent one sample of each available device. Any variation in device geometry due to manufacturing variability or other defects cannot be captured by this dataset. Device midlines were computed as the mean axial position of the 3D scanned geometry. This midline may not represent the true location of the interior metal ring, which gives the annuloplasty device its stiffness.

## Ethics Statement

The authors have read and follow the ethical requirements for publication and confirm that the current work does not involve human subjects, animal experiments, or any data collected form social media platforms.

## CRediT authorship contribution statement

**Collin E. Haese:** Data curation, Writing – original draft, Visualization. **Mrudang Mathur:** Methodology, Software, Formal analysis, Investigation, Writing – review & editing. **Marcin Malinowski:** Methodology, Software, Resources, Writing – review & editing, Supervision. **Tomasz A. Timek:** Resources, Funding acquisition. **Manuel K. Rausch:** Conceptualization, Methodology, Software, Formal analysis, Investigation, Writing – review & editing, Supervision, Funding acquisition.

## Data Availability

Tricuspid Annuloplasty Ring 3D Scans and Geometries (Original data) (Texas Data Repository).
